# Comparison of image quality of breast-specific positron emission tomography between opposite-type and ring-shaped systems: insights from Phantom and clinical studies in a Japanese multicenter trial

**DOI:** 10.1007/s12149-026-02165-5

**Published:** 2026-02-07

**Authors:** Yoko Satoh, Satoe Aoyama, Koji Itagaki, Yuka Naoi, Kanae K. Miyake, Masako Kataoka, Yoshitaka Inui, Hiroki Nosaka, Shigeki Ito, Yuichi Inaoka, Kenta Miwa, Kazunori Kubota, Masaki Uno, Yuki Kato, Kohei Hanaoka

**Affiliations:** 1 Imaging Center, Fujita Medical Innovation Center Tokyo, Haneda Innovation City Zone A, Hanedakuko 1-1-4, Ota, Tokyo Japan; 2 Clinical Imaging Center for Healthcare, Nippon Medical School, Tokyo, Japan; 3 Division of Clinical Radiology Service, Kyoto University Hospital, Kyoto, Japan; 4 Department of Advanced Imaging Research, Kyoto University Graduate School of Medicine, Kyoto, Japan; 5 Preemptive Medicine and Lifestyle-related Disease Research Center, Kyoto University Hospital, Kyoto, Japan; 6 Department of Radiology, Fujita Health University Hospital, Aichi, Japan; 7 Department of Radiological Technology and Science, Ibaraki Prefectural University of Health Science, Ibaraki, Japan; 8 Mirai Imaging Inc., Fukushima, Japan; 9 Medical Systems Division, Shimadzu Corporation, Kyoto, Japan; 10 Department of Radiological SciencesSchool of Health Sciences, Fukushima Medical University, Fukushima, Japan; 11 Department of Radiology, Dokkyo Medical University Saitama Medical Center, Saitama, Japan; 12 Division of Positron Emission Tomography, Institute of Advanced Clinical Medicine, Kindai University, Osaka, Japan

**Keywords:** Breast PET, Positron emission mammography (PEM), Dedicated breast PET (dbPET), Image quality, Acquisition time

## Abstract

**Objective:**

This study aimed to directly compare the imaging performance of the opposite-type positron emission mammography (PEM), first-generation photomultiplier tube (PMT)-based ring-shaped dedicated breast PET (dbPET1), and second-generation silicon photomultiplier (SiPM)-based dbPET (dbPET2) using clinical imaging protocols, and determine the requisite acquisition conditions for achieving comparable depiction of breast lesions across systems.

**Methods:**

A cylindrical phantom with four spheres (diameter: 3–10 mm) was prepared with sphere-to-background ratios (SBRs) of 2:1, 4:1, and 8:1, based on clinical images. The phantom was scanned for 10 min in list mode with the spheres at the center and periphery of each detector and reconstructed at 1–10 min. Visual and quantitative evaluations were performed using the coefficient of variation of the background (CV_BG_), detection index (DI), and contrast recovery coefficient (CRC). Representative clinical images of three lesion types, namely, mass uptake near the nipple, mass uptake close to the chest wall, and non-mass uptake, were also assessed using visual evaluation and the tumor-to-background ratio (TBR).

**Results:**

Phantom images with SBRs of 2:1 and 4:1 did not sufficiently visualize the small spheres; therefore, an 8:1 ratio was chosen for the analysis. dbPET was capable of visualizing smaller spheres compared with PEM. At the periphery, image quality was reduced for all systems, while all systems were able to identify spheres ≥ 7.5 mm in diameter at a contrast ratio of 1:8 under clinical imaging protocols. The DI decreased with shorter acquisition time, while the CRC remained relatively stable. The CV_BG_ increased, especially in dbPET2. Clinical evaluation confirmed the acquisition-time dependence of image quality in both PEM and dbPET systems, providing practical insight into their appropriate use under routine clinical conditions. TBR analysis supported the consistency between the phantom study results and the clinical evaluations.

**Conclusions:**

This study demonstrated that all breast-specific PET systems can achieve image quality capable of identifying sub-centimeter lesions within clinically feasible scan times. These findings provide the foundation for harmonizing protocols across systems and optimizing their clinical application in breast cancer diagnosis.

## Introduction

Breast-specific positron emission tomography (breast PET) was developed to overcome the limitations of conventional whole-body PET in small breast cancer (BC) detection, such as insufficient spatial resolution for delineating small intramammary lesions [1, 2]. By placing detectors in close proximity to the breast, breast PET has achieved higher spatial resolution and sensitivity than whole-body PET, enabling a more reliable visualization of primary breast lesions. The two main modalities are positron emission mammography (PEM), which employs two flat-panel detectors arranged in opposition with the breast lightly held between them while the patient is seated, and ring-shaped dedicated breast PET (dbPET), in which the patient lies prone, with the breast hanging through an opening in the bed into the cylindrical detector [3–5]. In addition to their high spatial resolution, breast PET systems offer several practical advantages over whole-body PET/CT, including a smaller installation footprint, lower power consumption, and simpler maintenance because no CT component is required. Because they do not use CT for attenuation correction or image reconstruction, they also provide lower radiation exposure.

In Japan, breast PET received public insurance coverage in July 2013, along with whole-body PET-CT scans. In the same year, the Japanese Society of Nuclear Medicine (JSNM) published the first edition of the Breast-Specific PET Clinical Practice Guidelines to support the appropriate use of breast PET. Thereafter, the increase in the number of breast PET scanners in Japan and their widespread clinical application, and the concurrent accumulation of scientific evidence, led to the publication of a revised edition of these guidelines in 2019 [6]. Recent nationwide survey data report 15 dedicated breast PET systems installed in Japan [7]. The survey relies on voluntary institutional responses; hence, the reported numbers may not fully reflect all existing installations. To complement these data, manufacturer-based estimates suggest that approximately 25 breast PET systems (3 PEM and 22 dbPET systems) are currently in clinical use in Japan. These guidelines provide recommendations for the appropriate clinical use of two different types of breast PET systems: PEM and dbPET. However, they did not address the technical aspects of either device, particularly the comparison of image quality between the two systems or their standardization.

Currently, PEM and dbPET systems manufactured domestically are in clinical use in Japan. In recent years, next-generation dbPET systems have been introduced, and the need for harmonization of image quality across different breast PET systems has become increasingly important.

We previously reported a comparison between two generations of dbPET systems, focusing on detector performance and post-filter optimization [8]; however, that work did not include PEM devices nor address phantom designs suitable for cross-system evaluation. The present study expands upon that earlier investigation by incorporating a PEM scanner and by assessing a breast-specific phantom configuration that enables unified evaluation across different breast PET architectures.

To the best of our knowledge, no previous study has directly compared PEM and dbPET systems under clinically relevant imaging conditions using a unified phantom and clinical evaluation framework. The objective of this study was to identify acquisition conditions that allow comparable visualization of similarly small BC lesions across PEM and dbPET.

## Methods

### Breast PET scanners

**Fig. 1:**
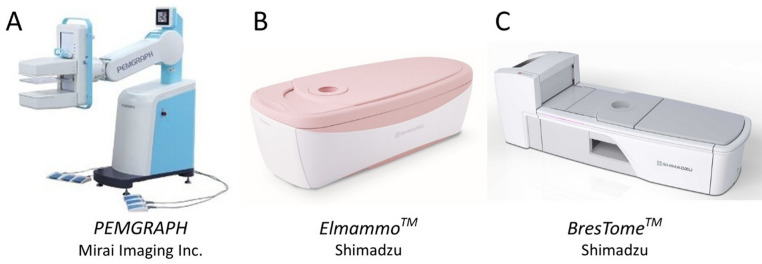
Japanese manufactured breast-specific PET scanners. (**a**) PEM: *PEMGRAPH* (Mirai Imaging Inc.). (**b**) dbPET1: *Elmammo Avant Class* (Shimadzu). (**c**) dbPET2: *BresTome* (Shimadzu). The detector position of “c” is movable, facilitating its application as a brain PET scanner as well (the photograph shows the breast position). Abbreviation: PET, positron emission tomography; PEM, positron emission mammography; dbPET1, photomultiplier tube–based dedicated breast PET; dbPET2, silicon photomultiplier–based dedicated breast PET

Three dedicated breast PET systems were evaluated in this study: opposite-type PEM, photomultiplier tube-based dbPET (dbPET1), and silicon photomultiplier-based dbPET (dbPET2) (Fig. 1).

### PEM (PEMGRAPH, Mirai imaging Inc., Japan)

The PEM system is composed of an opposite-type device equipped with two flat-panel detectors arranged in a face-to-face configuration, enabling high-resolution imaging of the soft-compressed breast (Fig. 1a). Previous studies have described its basic design and clinical performance, demonstrating its utility for detecting small BCs [9, 10]. PEM has a larger defection of the lines of response (LOR) compared with dbPET scanners, in which cylindrical detector arrays are placed around the breast. Therefore, usually only one direction of maximum intensity projection (MIP) is adopted per acquisition (not tomographic images). In this study, the images were reconstructed using the 3D-Maximum Likelihood Expectation Maximization algorithm with eight iterations and an anti-aliasing filter with dead time, random and decay correction, and no attenuation correction. Based on a phantom scan with known ^18^F- fluorodeoxyglucose (FDG) radioactivity, the pixel values of clinical PEM images were converted into standardized uptake values (SUVs).

### dbPET1 (Elmammo Avant Class™, Shimadzu Corporation, Japan) and dbPET2 (BresTome™, Shimadzu Corporation, Japan)

The dbPET system is equipped with a cylindrical detector module arrangement, enabling high-resolution imaging of the breast while it is hanging due to gravity [5, 11]. Because dbPET scanners have fewer missing LORs than PEM scanners, both mediolateral and craniocaudal MIPs as well as cross-sectional images are usually used for diagnosis per acquisition. In dbPET, pixel values are converted to the SUV through attenuation correction using a µ-map, which is obtained by detecting the skin surface from the emission scan data and assuming the breast is a homogeneous absorber.

dbPET1 is an earlier-generation ring-type dbPET system that employs conventional photomultiplier tubes, and dbPET2 is a next-generation ring-type dbPET system incorporating digital silicon photomultipliers, offering better sensitivity and spatial resolution compared with dbPET1(Fig. 1B and C) [5, 12]. In particular, the detector ring diameter of dbPET2 is larger than that of dbPET1, a design adopted to also accommodate brain imaging. Although a larger detector diameter generally reduces spatial resolution and count sensitivity in PET systems, the previous report demonstrated that the overall image quality of dbPET2 is equivalent to or slightly superior to that of dbPET1, likely owing to the use of SiPM detectors and TOF capability [8].

The primary specifications of these three systems are summarized in Table 1. Further technical details can be found in previous studies [8].

**Table 1:** Main specifications of the three breast-specific PET systems evaluated in this study

System type	PEM	dbPET1	dbPET2
Scanner Name	PEMGRAPH	Elmammo Avant Class™	BresTome™
Vendor	Mirai Imaging Co., Ltd.	Shimadzu	Shimadzu
Detector shape	Opposite plate-type	Ring- type	Ring- type
Detector aperture	100–250 mm^*^ (gap, plate)	φ195 mm (circ.)	φ300 mm (circ.)
FOV (mm)	202.4 × 140.8 (rect.)×100–250 mm^*^	φ182 (circ.)×103	φ264 (circ.) ×162
Material of crystal	LuAG	LGSO	LGSO
Crystal size (mm^3^)	1.5 × 1.5 × 11	1.44 × 1.44 × 18	2.1 × 2.1 × 15
Photo device	PMT	PMT	SiPM
Number of DOI layers	1	4	1
TOF Technology	Not available	Not available	Available
Matrix size	254 (X)×176 (Y)	236 (X)×236 (Y)×132 (Z)	240 (X)×240 (Y)×148 (Z)
Pixel size (mm^2^)	0.8	0.78	1.1
Spatial resolution at 5 mm offset from the center of the FOV (mm)^⁑^	up to 2.0	up to 1.5	from to 1.0 to 2.5
Sensitivity at 0 cm in the center of the FOV (cps/kBq)^⁑^	0.005	from 0.05 to 0.09	from 0.06 to 0.11

^*^Adjustable in 25 mm increments^, ⁑^NEMA NU 4–2008 ^22^Na source

**Abbreviations**: PET, positron emission tomography; PEM, positron emission mammography; dbPET, dedicated breast PET; FOV, field of view; rect., rectangular; circ., circular; LuAG, lutetium aluminum garnet; LGSO, lutetium gadolinium oxyorthosilicate; PMT, photomultiplier tube; SiPM, silicon photomultiplier; DOI, depth-of-interaction; TOF, time of flight; NEMA-NU, National Electrical Manufacturers Association – NU standard; cps/Bq, counts per second per becquerel

### Phantom preparation

**Fig. 2:**
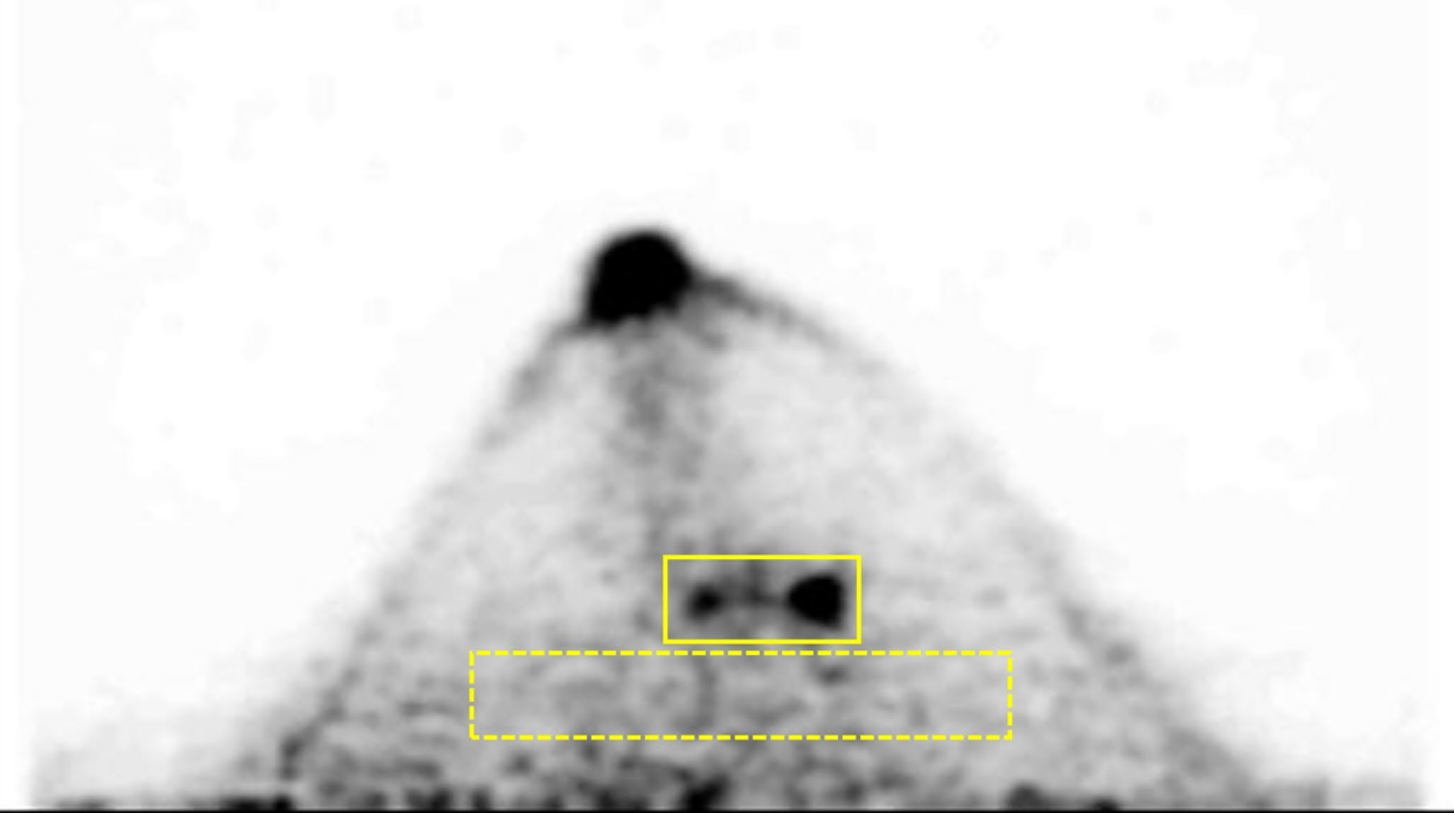
Example region-of-interests placement for determining the SBR used in the breast-specific PET phantom study. The rectangular region-of-interests for the breast lesion (solid line) and background breast tissue (dashed line) are placed on the dbPET2 transaxial image, for example. The SBR used in the phantom study was defined based on the radioactivity ratios obtained from these measurements. Abbreviation: SBR, sphere-to-background ratio; PET, positron emission tomography; dbPET, dedicated breast PET

A cylindrical phantom containing four hot spheres was used for the comparison of the breast PET scanners. In order to detect BC lesions with a diameter of 5 mm, which corresponds to T1a in the Union for International Cancer Control (UICC) TNM classification, spheres of four different sizes (3, 5, 7.5, and 10 mm) were adopted. For PET image evaluation based on phantom studies, the sphere-to-background radioactivity concentration ratio (SBR) is commonly set at 8:1. However, most previous studies were designed for whole-body PET scanners, lacking verification of the suitability of this SBR for breast PET. Therefore, in this study, the SBR was determined by measuring the radioactivity concentration of the breast lesion and background mammary gland on the clinical breast PET images of patients with BC or suspected BC (Fig. 2). A total of 68 breast PET images (PEM: 20, dbPET1: 22, dbPET2: 26) were analyzed. The activity concentrations (Bq/mL) were obtained using the 2D-region of interests (ROIs) placed on the breast lesion and the background mammary gland at the same axial level, avoiding pectoral muscle and noise at the edge of the ROI. The lesion-to-background radioactivity concentration ratios derived from these measurements were used to define the SBR for the phantom study.

### Phantom setup

**Fig. 3:**
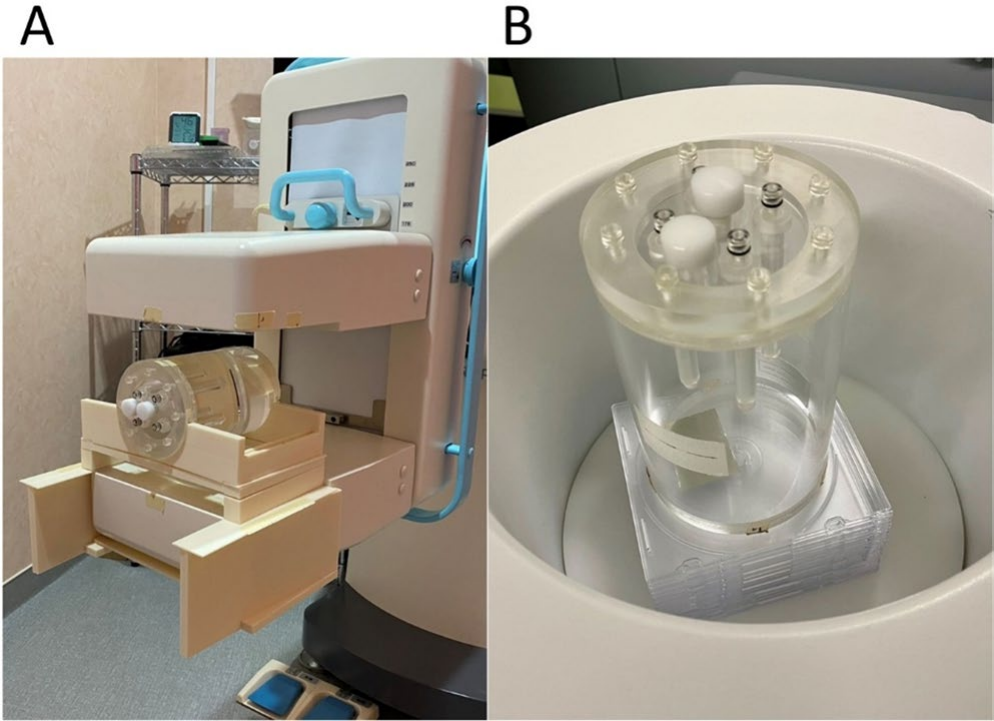
Placement of the cylindrical phantom on the breast-specific PET scanners The cylindrical phantom was placed on the PEM detector using a dedicated stand (**a**), and in the center of the dbPET detector with height adjusted by spacers (**b**). Abbreviation: PET, positron emission tomography; PEM, positron emission mammography; dbPET, dedicated breast PET

For the PEM system, a cylindrical phantom was positioned between the two flat-panel detectors, and a custom-made acrylate–styrene acrylonitrile stand was used to prevent rolling (Fig.3). For dbPET1 and dbPET2, the phantom was placed directly within the ring-shaped detector bore. When the position of the hot spheres needed to be changed, the phantom was elevated with spacers to adjust the vertical alignment.

### Data acquisition

Based on previous studies, the phantom was placed with four hot spheres arranged in the center of the field of view (FOV) (*center*) and 2 cm inside the edge of the detector (*periphery*) of each scanner [13, 14]. A custom-made acrylate–styrene–acrylonitrile stand was prepared to hold the cylindrical phantom on the PEM detector. The phantom prepared in each SBR was imaged for 10 min in list-mode at two positions (*center* and *periphery*).

### Image reconstruction

All the breast PET images were reconstructed using the clinical conditions for each scanner, which were determined based on the results of previous studies and clinical experience [8, 9, 12]. However, the acquisition time can vary in routine clinical practice depending on the patient’s condition. To determine the minimum acquisition time required for diagnosis, the phantom images were reconstructed using full data (10 min) and short-time acquisitions (1, 3, 5, and 7 min) by dividing the list-mode data. The reconstruction conditions specific to each scanner are summarized in Table 2.

**Table 2:** Main reconstruction parameters of the three breast-specific PET systems evaluated in this study

System type	PEM	dbPET1	dbPET2
Iterative reconstruction	3D-MLEM	3D-DRAMA	3D-DRAMA
Scatter correction	Not available	Convolution Subtraction	True estimate subtraction
Attenuation correction	Not available	Uniform attenuation map with object boundaries obtained from emission data	Uniform attenuation map with object boundaries obtained from emission data
Iterations/Subsets	8/1	1/128	1/100
Post filter	MRP	Median, Gaussian	Median, Gaussian, NLM
SUV conversion	Available	Available	Available

Abbreviations: PET, positron emission tomography; 3D, three-dimensional; ML-EM, maximum likelihood-expectation maximization; DRAMA, dynamic row-action maximum likelihood algorithm; MRP, Modified Ramp; NLM, non local means

### Analysis of Phantom image quality

All transverse phantom images that lined the largest cross-section of the hot spheres were displayed in inverse grayscale, with SUVs ranging from 0 to 4. In dbPET, all four hot spheres were displayed as a single transverse image, whereas in PEM, two spheres were displayed per image, resulting in two images covering all four spheres. First, two nuclear medicine physicians and two nuclear medicine technologists, all blinded to the SBR settings, visually evaluated the detectability of the hot spheres in the 10-min reconstructed images. A sphere was considered visually detectable when it appeared as a localized focal hotspot clearly distinguishable from background noise at the expected sphere position. This visual assessment served as the reference standard for sufficient detectability. Subsequently, the full-duration and short-duration images were compared visually and quantitatively to determine the minimum acquisition time at which comparable detectability was maintained.

For quantitative evaluation, circular regions of interest (ROIs) with the same diameter as the sphere were placed on the spheres in the phantom images. Additionally, eight ROIs with a diameter of 10 mm were placed in the background, and the coefficient of variation of the background (CV_BG_), detection index (DI), and contrast recovery coefficient (CRC) were calculated using the following formulae:

$$\:\mathrm{C}\mathrm{V}_{\mathrm{B}\mathrm{G}\:}=\frac{{SD}_{BG,10mm}}{{C}_{BG,10mm}},$$

where SD_BG,10 mm_ is the standard deviation of the background ROI for a 10-mm diameter circle, and C_BG,10 mm_ is the average of the background ROI values for a 10-mm diameter circle.

$$\:\mathrm{D}\mathrm{I}\:=\:\frac{{C}_{H,5mm;max}-{C}_{BG,10mm;mean}}{{SD}_{BG}},$$

where C_H,5mm;max_ is the maximum SUV (SUV_max_) of the ROI for a 5-mm diameter sphere and C_BG,10mm;mean_ is the average of the background ROIs for a 10-mm diameter circle

$$\:\mathrm{C}\mathrm{R}\mathrm{C}\:=\frac{\left({C}_{H;max}/{C}_{BG:mean}\right)-1}{\left({a}_{H}/{a}_{BG}\right)-1},$$

where a_H_ and a_BG_ represent the activity concentrations in the hot sphere and background, respectively.

As standardized quantitative criteria for breast PET have not been established, the use of CRC, CV_BG_, and DI in this study was informed by previous phantom investigations of whole-body PET/CT, where these parameters are commonly used to evaluate image quality and detectability [15, 16]. As absolute recommendations for CV_BG_, CRC, and DI in breast PET have not been established, the impact of acquisition time on these parameters was evaluated. These quantitative findings were then integrated with visual assessments to determine the minimum acquisition time for each scanner.

### Human imaging

This study was approved by the Medical Research Ethics Review Committee of the Fujita Health University (HM25-186). All procedures were performed in accordance with the ethical standards outlined in the 1964 Declaration of Helsinki and its subsequent revisions. Based on the retrospective observational study design, informed consent was waived for all patients.

Patients fasted for at least 6 h before the ^18^F-FDG (3.7 MBq/kg) injection. Following a whole-body PET/CT scan, the breasts were scanned separately on each side, with breast PET commencing approximately 90 min after the administration of ^18^F-FDG. Breast PET images were also reconstructed using clinical conditions. Full and short-acquisition-time clinical PET images of representative cases scanned using each scanner were reviewed and discussed, along with the results of the phantom studies.

Representative BC images were presented using three breast PET scans and other modalities to compare the characteristics of the three breast PET systems. These cases were selected to demonstrate the typical imaging features of three different types of lesions based on previous studies [17, 18]: (i) mass uptake near the nipple, (ii) mass uptake slightly near the chest wall, and (iii) non-mass uptake (NMU).

In accordance with the phantom study, short-time acquisition images were reconstructed from the list-mode data of the clinical NMU lesions. For quantitative evaluation, SUV_max_ and tumor-to-background ratio (TBR) were calculated using the following equations:

$$\:\mathrm{T}\mathrm{B}\mathrm{R}\:=\frac{{SUV}_{\:T,max}}{{SUV}_{BG,mean}},$$

where SUV_T,max_ is the SUV_max_ of the tumor (BC lesion) and SUV_BG,mean_ is the mean value of the largest circular ROI placed on the background to avoid breast tumors, skin, and noise at the edge of the FOV, respectively.

## Results

### Phantom study

**Fig. 4:**
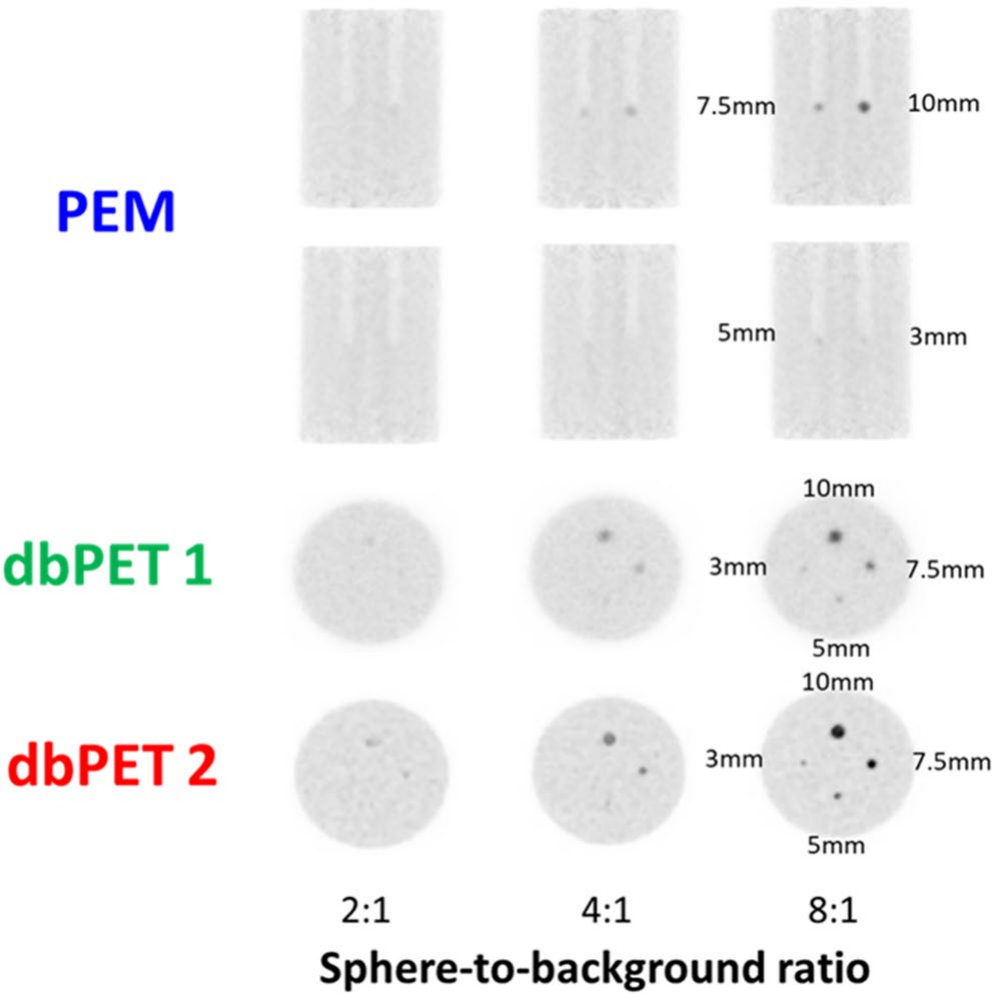
Phantom images obtained with the three breast-specific PET systems (PEM, dbPET1, and dbPET2, from top to bottom). The hot spheres were located at the center of the detector and acquired for 10 min at an SBR 2:1, 4:1, and 8:1.Abbreviations: PET, positron emission tomography; SBR, sphere-to-background ratio; PEM, positron emission mammography; dbPET, dedicated breast PET

Based on clinical PET image measurements of 68 patients with BC, the background radioactivity concentration of 2.56 kBq/mL and SBRs of 2:1, 4:1, and 8:1 were determined. The phantom images with SBRs of 2:1 and 4:1 did not sufficiently visualize the hot spheres (Fig. 4). Accordingly, phantom images with an SBR of 8:1 were used for quantitative evaluation.

**Fig. 5:**
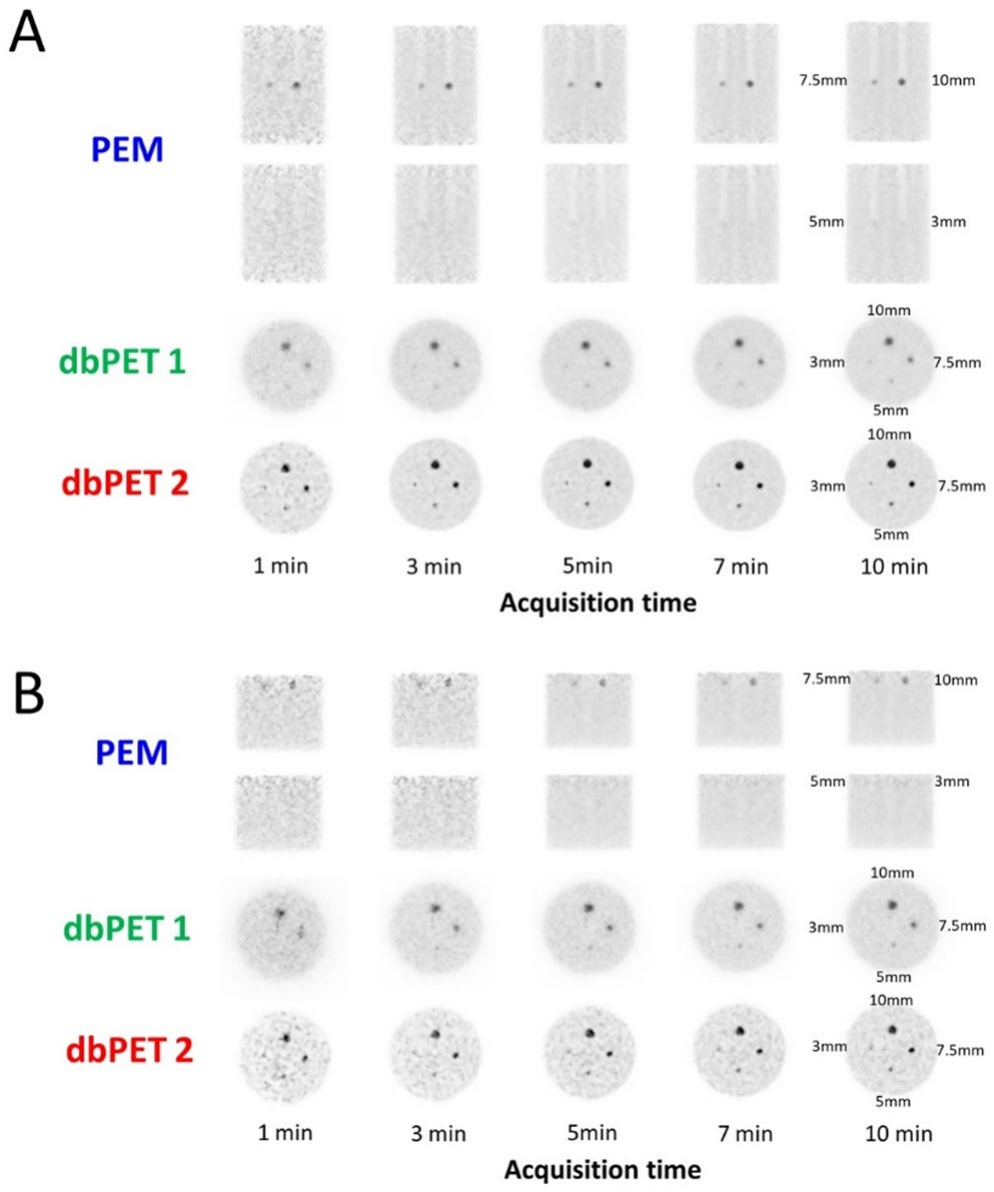
Breast-specific PET images acquired at 1, 3, 5, 7, and 10 min in a phantom with an SBR of 8:1. The hot spheres were placed at the center (**a**) and periphery (**b**) of the detectors. In this study, ‘periphery’ was defined as 2 cm inside the detector edge, as described in the Methods section. Abbreviations: PET, positron emission tomography; SBR, sphere-to-background ratio

The phantom images acquired at 1, 3, 5, 7, and 10 min using breast PET were reconstructed from 10 min of list-mode data from a phantom with an SBR of 8:1 (Fig. 5). We found that dbPET had the ability to better visualize smaller spheres than PEM and that dbPET2 could visualize spheres more clearly compared with dbPET1. The detailed reader-based detectability outcomes for each sphere size and acquisition time are summarized in Table 3. These results explicitly show the minimum acquisition time required for sphere identification under center and peripheral conditions, supporting the visual trends described above. In addition, the ability to visualize the spheres was lower at the periphery than at the center for all the scanners.

**Table 3:** Minimum acquisition times for visual detectability of hot spheres in the Phantom study (SBR 8:1)

Sphere diameter	Location	PEM	dbPET1	dbPET2
5 mm	Center	Not visible	1 min	1 min
	Periphery*	Not visible	5 min	1 min
3 mm	Center	Not visible	10 min	3 min
	Periphery	Not visible	Not visible	5 min

Notes: “Visible” indicates consensus identification by two nuclear medicine physicians and two PET technologists. *Periphery = 2 cm inside the detector edge

Visual assessment of the short-time acquisition images showed that 7.5-mm and 10-mm diameter spheres were visualized in all images acquired in 1 min. Even with a 10-min acquisition time, the PEM system could not visualize spheres measuring 5 mm or smaller, and the dbPET1 system also had difficulty visualizing 3-mm spheres at the periphery. In contrast, a 3-mm sphere could be identified at the periphery at 5-min acquisition using dbPET2.

**Fig. 6:**
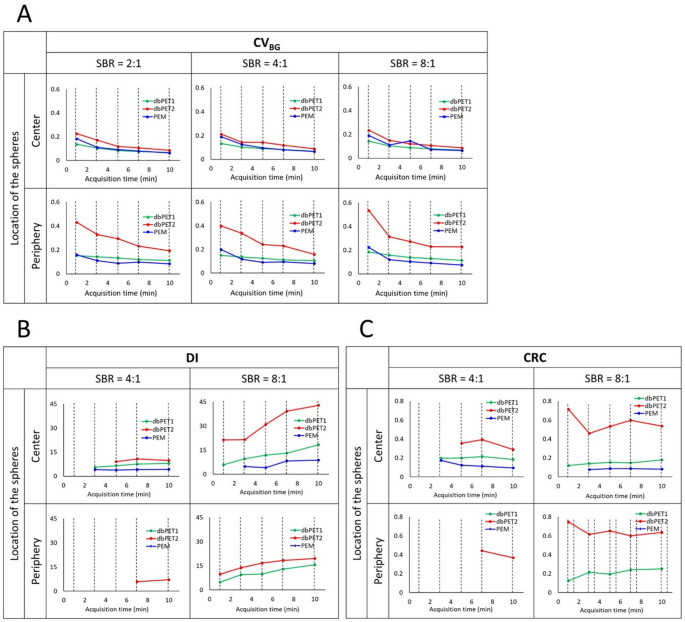
Quantitative evaluation of phantom images scanned with three breast-specific PET scanners. The hot spheres were located at the center of the detector (*center*) and 2 cm inside from the edge of the detector (*periphery*), and the images were reconstructed from 1, 3, 5, 7, and 10-min acquisition data at three different sphere-to-background radioactivity concentration ratios (2:1, 4:1, and 8:1). The DI (**B**) and CRC (**C**) were calculated for the 5-mm spheres that could be visually identified.Abbreviations: PET, positron emission tomography; DI, detection index; CRC, contrast recovery coefficient

Quantitative evaluation showed that CV_BG_ was largely stable across different SBR conditions; however, it increased with shorter acquisition times, a trend that was most pronounced with dbPET2 at the periphery (Fig. 6). The DI and CRC were calculated for the 5-mm spheres that could be visually identified. The 5-mm sphere was not identified in any image at an SBR of 2:1. The shorter the acquisition time, the smaller the DI; however, the CRC value showed little change with respect to the acquisition time. The DI and CRC decreased in the following order: dbPET2, dbPET1, and PEM. In the PEM image, no 5-mm spheres were identified in the periphery.

The results of the phantom study suggested that, even with breast PET, it was difficult to visualize BC with a low uptake approximately twice that of the background and a size of 10 mm or less. Furthermore, considering the identification of BC with a contrast against the background lesser than 8:1, the minimum acquisition time was 5 min, and 7 min was considered optimal if possible.

### Clinical case review

Three representative clinical cases were included as illustrative examples rather than systematic clinical evidence. Cases were selected according to the following predefined criteria: (i) pathologically confirmed BC; (ii) primary tumors classified as T1 or smaller, for which breast PET has high clinical utility; (iii) mass uptake located centrally or peripherally, selected to reflect the “center” and “periphery” conditions of the phantom study; and (iv) one non-mass uptake lesion, included because breast PET has been shown to provide high contrast for such lesions in prior studies [18].

**Fig. 7:**
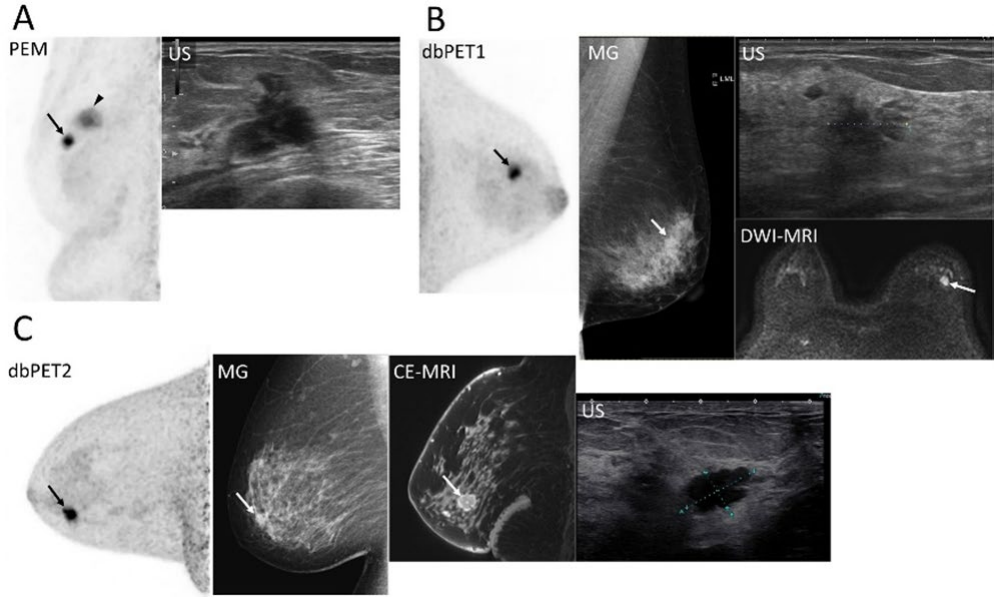
Representative breast-specific PET images for a mass uptake closer to the nipple and inter-modality differences.The primary breast cancer lesions are indicated by arrows. All PET images are displayed using an SUV scale ranging from 0 to 5. (**a**) A 71-year-old woman with invasive ductal carcinoma (IDC, luminal A): PEM and ultrasonography. A DCIS is also shown by an arrowhead. (**b**) A 61-year-old woman with IDC (luminal B): dbPET1, mammography (MLO), ultrasonography, and non-contrast MRI (DWI). (**c**) A 50-year-old woman with IDC (triple negative): dbPET2, mammography (MLO), contrast-enhanced MRI, and ultrasonography. Abbreviations: PET, positron emission tomography; PEM, positron emission mammography; US, ultrasonography; IDC, invasive ductal carcinoma; DCIS, ductal carcinoma in situ; dbPET, dedicated breast PET; MG, mammography; MLO, mediolateral oblique; DWI, diffusion-weighted imaging; CE, contrast-enhanced

**Fig. 8:**
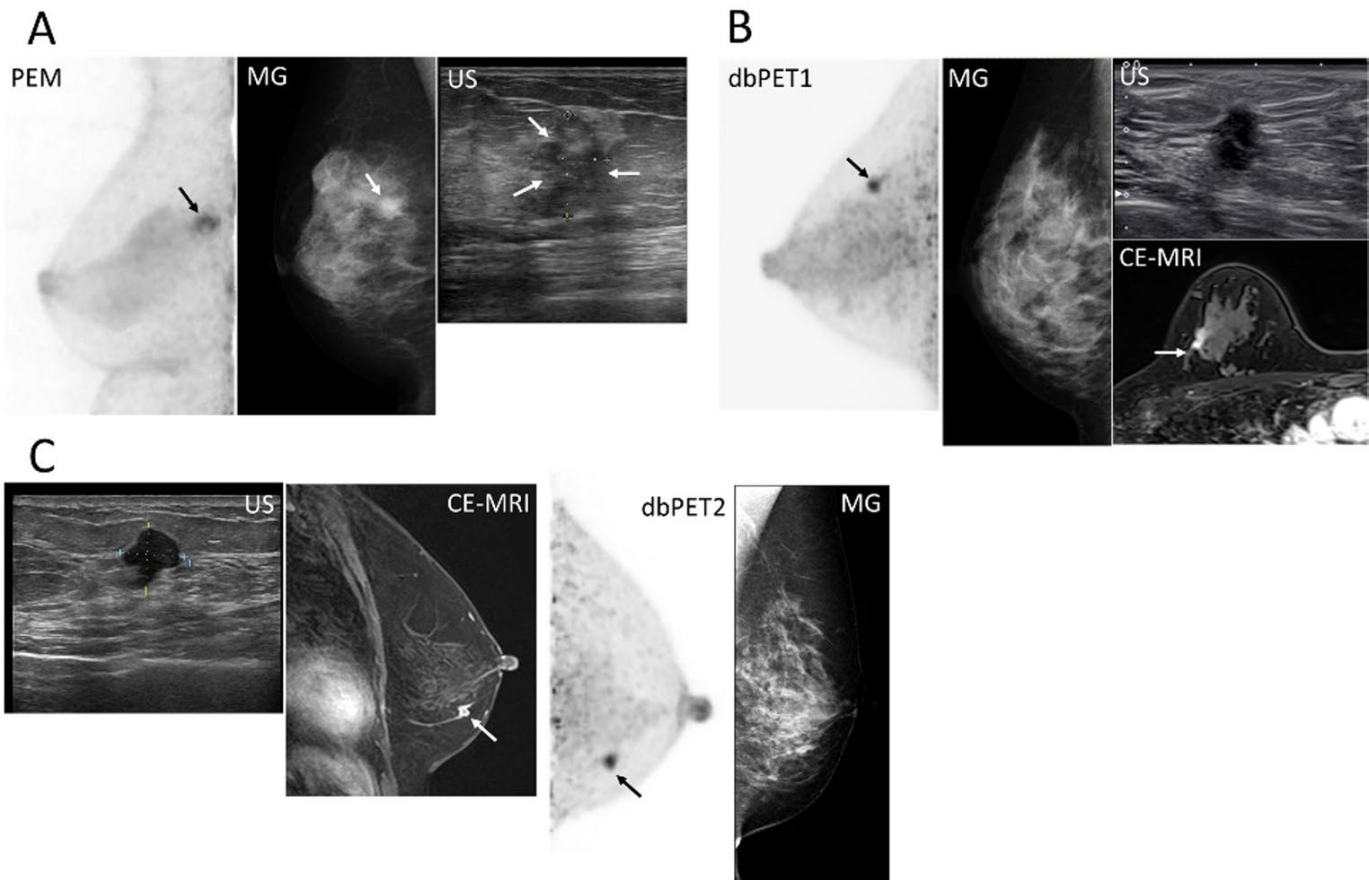
Representative breast-specific PET images for a mass uptake slightly closer to the chest wall and inter-modality differences. The primary breast cancer lesions are indicated by arrows. All PET images are displayed using an SUV scale ranging from 0 to 5. (**a**) A 44-year-old woman with invasive ductal carcinoma (IDC, Luminal A). (**b**) A 45-year-old woman with IDC (Luminal B). dbPET1, mammography (MLO), ultrasonography, and contrast-enhanced MRI. The arrow marks the primary lesion; mild peripheral noise is visible near the chest-wall side of the FOV. (**c**) A 56-year-old woman with IDC (Luminal B). dbPET2, mammography (MLO), ultrasonography, and contrast-enhanced MRI. The primary lesion is indicated by an arrow; a slight peripheral noise increase is observed. Abbreviations: PET, positron emission tomography; PEM, positron emission mammography; MG, mammography; MLO, mediolateral oblique; US, ultrasonography; IDC, invasive ductal carcinoma; FOV, field of view; dbPET, dedicated breast PET; CE, contrast-enhanced

**Fig. 9:**
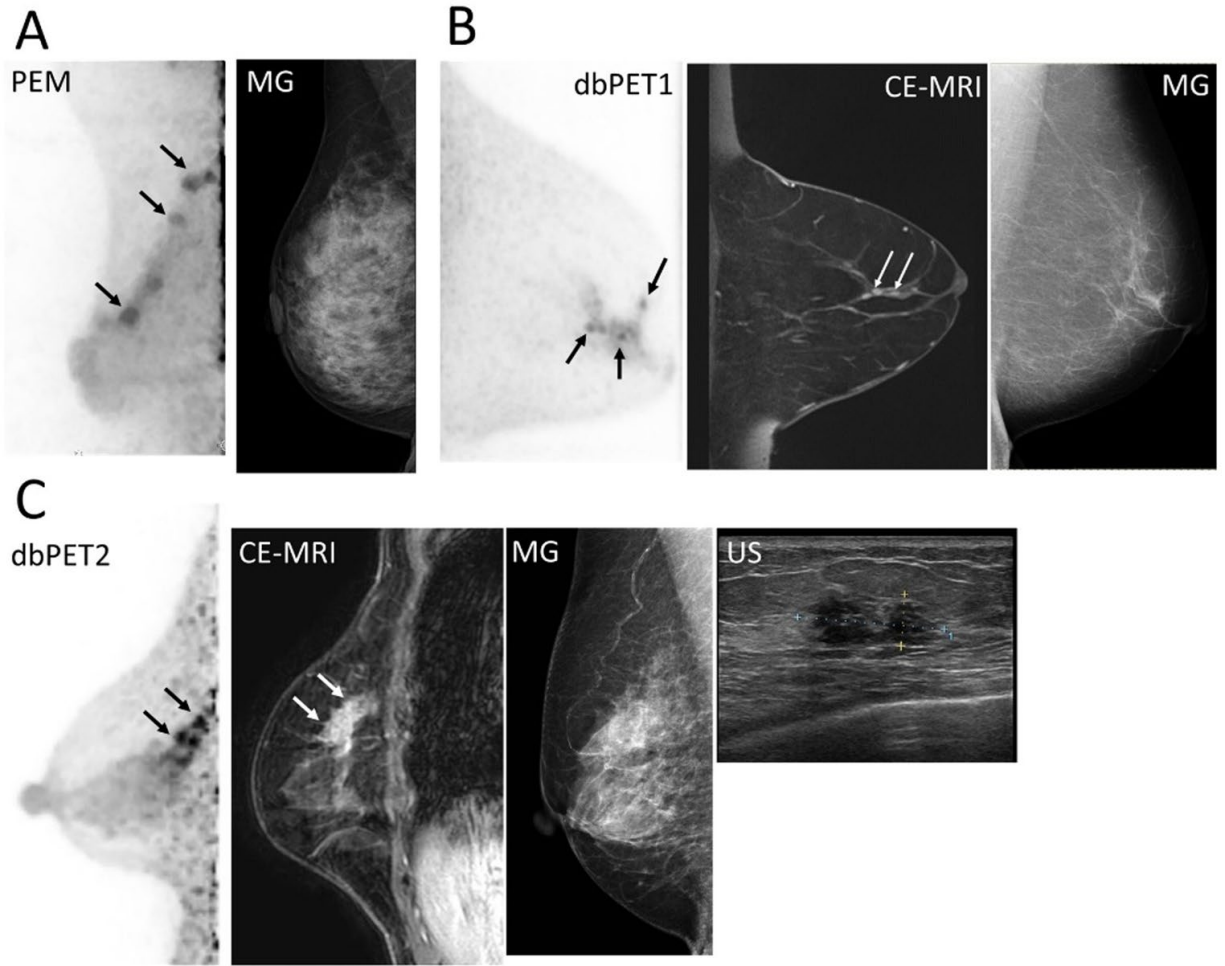
Representative breast-specific PET images for a non-mass uptake and modality differences. The primary breast cancer lesions are indicated by arrows. All PET images are displayed using an SUV scale ranging from 0 to 5. (**a**). A 42-year-old woman with invasive ductal carcinoma (IDC) and ductal carcinoma in situ (DCIS), luminal A**).** PEM and mammography (MLO). PEM was performed instead of MRI due to claustrophobia. (**b**). A 71-year-old woman with DCIS (intermediate type). dbPET1, contrast-enhanced MRI (delayed sagittal), and mammography (MLO). (**c**) A 50-year-old woman with triple-negative IDC. dbPET2, contrast-enhanced MRI, mammography (MLO), and ultrasonography. Abbreviations: PET, positron emission tomography; PEM, positron emission mammography; MG, mammography; MLO, mediolateral oblique; US, ultrasonography; IDC, invasive ductal carcinoma; DCIS, ductal carcinoma in situ; dbPET, dedicated breast PET; CE, contrast-enhanced

The first set of images presents BC lesions with mass uptake located near the nipple (Fig. 7). All three breast PET systems clearly visualized the lesions. However, the lesion margins appeared slightly sharper on images obtained with dbPET than on those obtained with PEM, which offered a slightly lower spatial resolution. This trend was consistent with the results of the preceding phantom study. The next set of cases presents BC images showing mass uptake closer to the chest wall (Fig. 8). All breast PET images visualized BC lesions, and the visual impression differed among scanners. In the PEM images, lesion conspicuity appeared reduced relative to the ring-type systems, a finding that aligned with the positional characteristics demonstrated in the phantom study. The third set of cases comprised non-mass uptake BC (Fig. 9). Breast PET visualized BC across all systems, while no corresponding abnormality was noted on mammography. In dbPET1 and dbPET2, the lesion location corresponded with that observed on contrast-enhanced MRI; PEM also depicted the lesion, although direct comparison with contrast-enhanced MRI was not available in this case.

**Fig. 10:**
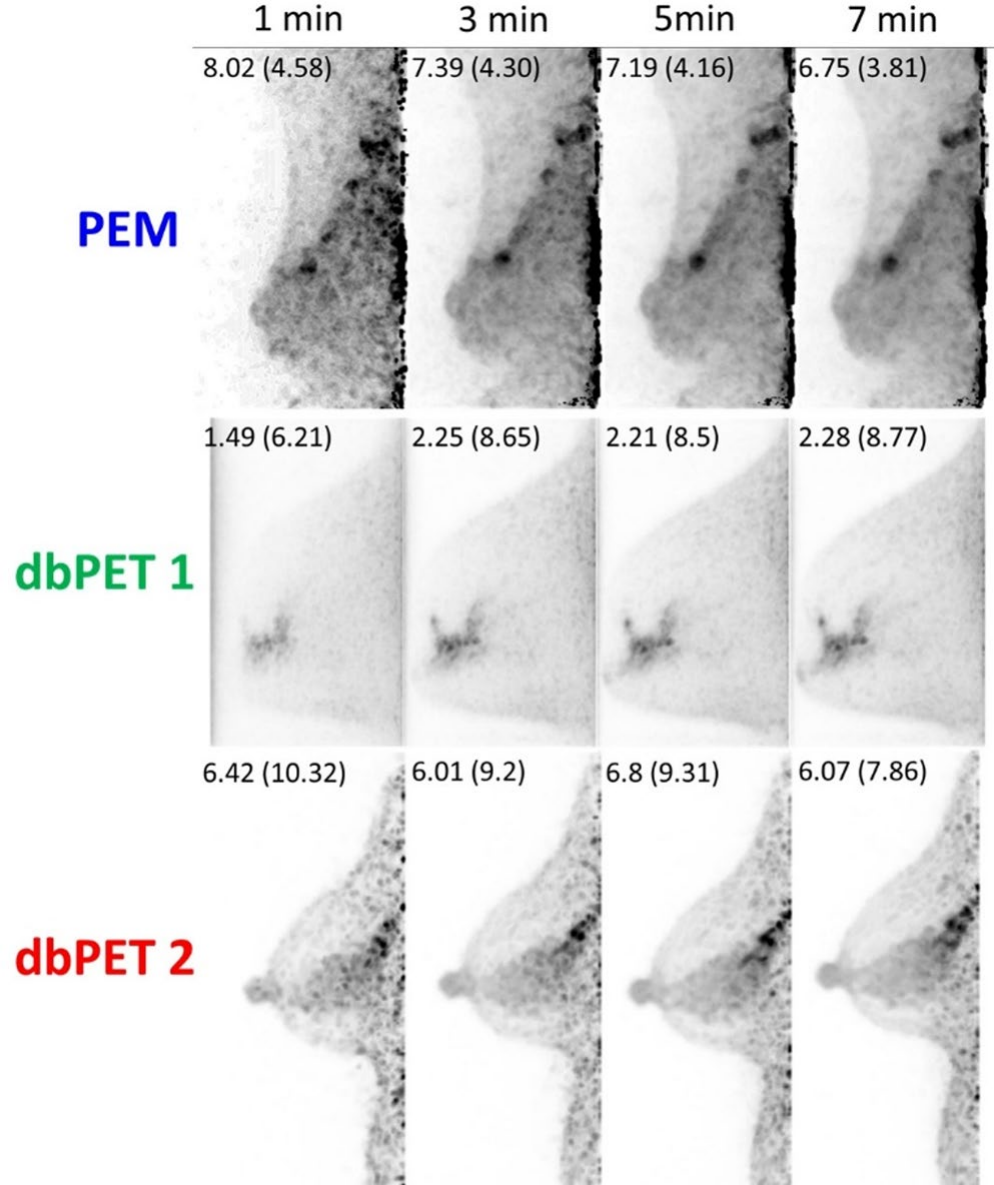
Short-time acquisition breast-specific PET images for a non-mass uptake obtained using three systems. Medio-lateral maximum intensity projection images acquired with PEM, dbPET1, and dbPET2 are shown up to 7 min. Short-time acquisition images were reconstructed from 1-, 3-, and 5-min subsets extracted from the 7-min list-mode data. The values displayed in each image represent the tumor SUV_max_ (tumor-to-background ratio). Abbreviations: PET, positron emission tomography; PEM, positron emission mammography; dbPET, dedicated breast PET

Additionally, for each breast PET scan, short-time acquisition images of NMU BCs were reconstructed based on the phantom study (Fig. 10). Visually, shorter acquisition times resulted in increased noise at the edge of the FOV near the chest wall in all breast PET images, a pattern consistent with the phantom findings and particularly marked in the PEM and dbPET2 images. In quantitative evaluation, both SUV_max_ and TBR increased with shorter acquisition times for PEM and dbPET2 showing BC lesions near the chest wall, while they decreased for dbPET1 showing a BC lesion near the nipple. The evaluation of clinical images reconstructed from the short-time acquisition data indicated that longer acquisition times yielded more stable image quality.

## Discussion

To our knowledge, no previous work has directly compared PEM and multiple generations of dbPET systems under clinically relevant imaging conditions. Unlike our previous study, which focused solely on dbPET systems, the present work newly includes a PEM scanner and evaluates a breast-specific phantom configuration to enable cross-system comparison [8]. This study aimed to identify conditions that yield comparable image quality across different scanner architectures; therefore, all images were reconstructed using clinical parameters routinely applied to each device. This approach was adopted because extensive optimization studies, particularly for dbPET, have already been reported and further fine-tuning of the reconstruction settings is neither practical nor clinically meaningful [18]. The acquisition duration is the most critical factor in clinical practice, since it directly affects both patient comfort—longer scans increase discomfort—and image quality—shorter scans compromise diagnostic reliability.

In this study, we directly compared the imaging performances of the opposite-type PEM and two generations of ring-type dbPET devices using both phantom and clinical data. Although PEM systems used in earlier studies required compression of the breast between the opposing plate-like detectors [19], the PEM scanner used in this study (PEMGRAPH, Mirai Imaging Inc.) does not employ compression. Instead, the breast is only lightly supported, and in routine clinical practice, the distance between the two plates is typically widened to ensure adequate inclusion of the chest wall within the FOV. Therefore, the phantom setup employed in this study would be consistent with the clinical use of the current PEM system. Although a deformable, breast-mimicking phantom would more closely approximate in vivo PEM imaging conditions, its use entails practical challenges, including limited mechanical durability, reduced reproducibility, and higher cost. Given that the clinical imaging of the same patient with multiple breast PET systems is not ethically feasible, a rigid cylindrical phantom was considered an acceptable compromise to enable standardized and repeatable cross-system evaluation in this study.

The phantom study demonstrated that dbPET systems, particularly dbPET2, achieved superior detectability of small spheres compared with PEM, while the image quality was consistently lower at the periphery of the detectors. Previous comparative studies of dbPET1 and dbPET2 have reported that SiPM-based systems show more noticeable noise near the detector edge [8]. A likely explanation is that dbPET2 has a substantially larger detector diameter, which increases the distance from the breast and reduces count sensitivity in the peripheral region. The larger diameter also makes the system more susceptible to spill-in of radioactivity from the torso, particularly myocardial uptake, which can influence reconstruction at the FOV boundary. In addition, edge-related limitations in normalization and scatter correction become more apparent in the small FOV of breast PET. Taken together, these factors are likely to contribute the increased noise observed at the detector periphery in dbPET2. Although CV_BG_ remained stable across scanners and acquisition times in most conditions, it increased at the periphery with dbPET2 as acquisition time became shorter, consistent with these peripheral noise characteristics. These findings were further supported by the clinical image review, which revealed similar trends across different lesion types.

Beyond these count-related and correction-related factors, detector geometry and structure may also influence image quality at the periphery of the detector. As a technical difference between dbPET1 and dbPET2, dbPET1 is equipped with a four-layer depth-of-interaction (DOI) detector, whereas dbPET2 incorporates time-of-flight (TOF) capability. At the detector periphery, where obliquely incident photons are more frequent, DOI information is likely to contribute, at least in part, to improved localization in the depth direction. However, because the present study was not designed to isolate the effect of DOI and multiple system characteristics differ between dbPET1 and dbPET2, the independent impact of DOI on peripheral image quality cannot be determined.

From a practical standpoint, these peripheral limitations directly affect the minimum acquisition time required for reliable imaging. Accordingly, acquisition times of ≥ 5 min for PEM and ≥ 3 min for dbPET could be considered acceptable for maintaining diagnostic quality. These differences correspond to variations in the defection rate of the LOR associated with the detector geometry of opposite-type and ring-shaped systems. In fact, at the periphery of the detector, where LOR deficiency was significant, deterioration in the PEM image quality was remarkable in the phantom study. In clinical practice, the feasibility of maintaining adequate image quality within practical acquisition times is particularly relevant. Based on our findings, for patients who have difficulty keeping the required scanning position, acquisition times of approximately 5 min for PEM and 3 min for dbPET may represent reasonable and practical options.

However, in the review of the clinical images, no disadvantage of PEM was observed in the visualization of BC close to the chest wall. PEM allows adjustment of the distance between the two plate-like detectors, enabling deeper inclusion of the chest wall within the FOV. Thus, PEM more readily visualized lesions close to the chest wall in the clinical examples, a finding that is consistent with its detector geometry. In contrast, dbPET has a fixed gantry diameter, which means that lesions close to the chest wall may fall outside the FOV, a limitation that persists even with the newer SiPM-based system [20]. Consequently, PEM may offer practical advantages in visualizing lesions near the chest wall, owing to its adjustable detector geometry, which reduces the likelihood of blind areas.

In 2019, the JSNM published a revised version of clinical practice guidelines for breast PET [6]. Compared with the first edition issued in 2013, the more recent guidelines include more clinical evidence, enhancing their coverage of clinical applications and safety considerations. However, because of significant differences in the geometric structures of the PEM and dbPET scanners, recommendations for standardizing image quality were not included. Recently, with the release of new-generation dbPET, the need for intersystem harmonization has become even more critical. The present study aimed to fill this gap by providing a comparative evaluation of PEM and two generations of dbPET under standardized phantom and clinical conditions. Importantly, the purpose of this investigation was not to determine which device is superior but rather to clarify how each system can be appropriately utilized to maximize patient benefit. Breast PET can provide valuable diagnostic information, irrespective of the scanner type, provided that the acquisition conditions are optimized. Our results emphasize that patients should not be disadvantaged simply because one type of breast PET system was used instead of another. Based on our findings, patients with BC scanned with different breast PET systems will benefit equally.

Contrast-enhanced breast MRI is considered the gold standard for evaluating the extent of the primary breast lesion. This study did not systematically compare breast PET with contrast-enhanced breast MRI. However, previous studies comparing these two modalities have demonstrated that their detection rates are equivalent or that breast PET exhibits higher specificity, if the lesion lies in the FOV [21–23]. On contrast-enhanced MRI, background parenchymal enhancement is marked in young women and often masks breast lesions; however, even in such patients, physiological FDG uptake in the background mammary gland has little impact on the detection of breast lesions. The use of MRI is restricted to patients who cannot receive contrast agents due to allergies, renal impairment, those with claustrophobia, or those with metallic implants (in their bodies). In such cases, breast PET may serve as a valuable alternative or complementary tool. At our institution, PEM is prioritized for patients who are unable to undergo contrast-enhanced breast MRI.

This study had a limitation. The number of clinical cases was relatively small. Thus, future studies with larger patient cohorts and a broader range of scanners are warranted to validate and generalize the present findings. Because this study focused on domestically developed systems, further studies investigating other breast PET platforms are important for broader generalization. Moreover, as body habitus and breast size vary across populations, international studies that include diverse ethnic groups are essential to fully establish the clinical utility of breast PET.

In conclusion, this multicenter phantom and clinical study demonstrated that opposite-type PEM and first- and second-generation dbPET systems are all capable of achieving diagnostic image quality for sub-centimeter lesions within clinically feasible acquisition times. Through direct comparison of three distinct detector geometries using clinically employed image reconstruction settings, differences in image characteristics among the systems were characterized, and acquisition times that may provide practical guidance for achieving acceptable image quality in routine clinical practice.
